# Five Digit Test in neuropsychological assessment of working memory in aged individuals: normative data

**DOI:** 10.1590/1980-5764-DN-2024-0141

**Published:** 2024-09-02

**Authors:** Juliana Francisca Cecato

**Affiliations:** 1 Universidade São Francisco, Departamento de Psicologia e Neuropsicologia, Itatiba SP, Brazil.; 2 Faculdade de Medicina de Jundiaí, Departamento de Clínica Médica, Ambulatório de Geriatria e Gerontologia, Jundiaí SP, Brazil.

**Keywords:** Memory Short-Term, Neuropsychology, Dementia, Psychometrics, Aging, Memória de Curto Prazo, Neuropsicologia, Demência, Psicometria, Envelhecimento

## Abstract

Five Digit Test (FDT) is an executive function assessment tool designed to be used across the lifespan, from children to the aged.

**Objective:**

To provide validity evidence for FDT in the neuropsychological assessment of working memory in the aged.

**Methods:**

A total of 100 subjects, aged between 56 and 86 years, representing both genders with varying levels of education, underwent a comprehensive clinical history and neuropsychological evaluation using FDT. The mean age of participants was 71.04 years, with 67.3% having intermediate education. Participants were categorized into two groups: Control Group and Cognitive Decline Group. To establish validity evidence, FDT scores (both time and errors) were correlated with the WAIS-III Digits scale. Spearman’s correlation coefficient and ROC curve methodology were employed to determine psychometric properties.

**Results:**

A significant and moderate negative correlation was evident between FDT Shifting (number of errors) and Digits score (rho=-0.51; p<0.0001), Direct Order (rho=-0.39; p<0.0001), and Indirect Order (rho=-0.46; p<0.0001). The area under the curve was higher for FDT Shifting (errors) (AUC=0.935) for a cutoff point greater than or equal to 5 points, compared to Digits (AUC=0.748).

**Conclusion:**

The assessment of the number of errors in FDT Shifting appears to be a statistically significant tool for evaluating working memory impairment in the aged.

## RESUMO

O Teste dos Cinco Dígitos (*Five Digit Test* — FDT) é um instrumento de função executiva para avaliação ao longo da vida, desde crianças até idosos.

**Objetivo:**

Apresentar evidências de validade para o FDT na avaliação neuropsicológica da memória operacional em idosos.

**Métodos:**

O total de 100 participantes, com idades entre 56 a 86 anos, de ambos os sexos, diferentes níveis de escolaridade, foi submetido a anamnese clínica detalhada e avaliação neuropsicológica por meio do FDT. A média de idade foi igual a 71,04 anos, 67,3% com escolaridade média. Os participantes foram divididos em dois grupos: Grupo Controle e com Declínio Cognitivo. Para verificar evidências de validade, os escores do FDT (tempo e erros) foram correlacionados ao subteste Dígitos da WAIS-III. O coeficiente de correlação de Spearman e a metodologia da Curva *Receiver Operating Characteristic* (ROC) foram utilizados para determinar as propriedades psicométricas.

**Resultados:**

Verificaram-se coeficientes de correlação negativa significativos e moderados entre o FDT Alternância (número de erros) e o escore do Dígitos (rho=-0,51; p<0,0001), Ordem Direta (rho=-0,39; p<0,0001) e Ordem Indireta (rho=-0,46; p<0,0001). A área sob a curva (AUC) apresentou-se no item FDT Alternância (número de erros) com maior valor (AUC=0,935) para um ponto de corte maior e igual a 5 pontos em comparação ao Dígitos (AUC=0,748).

**Conclusão:**

A avaliação do número de erros no item Alternância do FDT parece ser uma ferramenta estatisticamente significativa para avaliar o comprometimento da memória operacional em idosos.

## INTRODUCTION

Neuropsychological assessment plays an important role in promoting mental health and identifying changes that may occur in the aging process^1,2^. Particularly in the context of the aged, this assessment is an examination aimed at understanding and monitoring cognitive status, identifying potential declines, and implementing appropriate intervention strategies^3^. Several instruments are employed in the diagnostic investigation of the aged, including the Five Digit Test (FDT), a validated executive function test for the Brazilian population in 2015^4^. FDT is widely used in neuropsychological assessment, both for children suspected of neurodevelopmental disorders and for aged individuals suspected of neurocognitive disorders^4,5^. The information contained in the Technical Manual of application refers to the test as a non-verbal instrument, with its use applicable to different age groups, varying educational qualifications, and even individuals with atypical developmental or psychiatric conditions. According to the test authors, Sedó, de Paula and Malloy-Diniz^4^, the wide applicability of the test is due to its minimal execution requirement. In other words, the test is a cognitive function instrument based on minimal linguistic knowledge: reading digits from 1 to 5 and counting quantities from 1 to 5. According to the authors, this simplicity can represent a considerable advantage in today’s society, where increased occupational, commercial, and cultural exchanges leads to a growing diversity in the Brazilian population, including educational levels^4,5^. FDT was built based on universal symbols and a preschool-level vocabulary, making it applicable to an illiterate population or cases with minimal language knowledge, such as subjects who can only respond to the test without their mother tongue or primary dialect^4,5^. The application manual divides the normative correction tables of the instrument by age groups: 6 to 8 years; 9 to 10 years; 11 to 12 years; 13 to 15 years; 16 to 18 years; 19 to 34 years; 35 to 59 years; 60 to 75 years; and 76 years old or older. It disregards years of education^4^.

In the diagnostic investigation process of dementias, it is essential to correlate structural performance, through neuroimaging exams, with functional performance, as assessed by neuropsychological evaluation. It is noteworthy that macroscopic structural aging results in atrophy, with increased prominence between sulci and gyri. The prefrontal cortex, temporal cortex, and hippocampus experience volume reduction, accompanied by ventricular enlargement^6^. Microscopic changes, measured through specific exams, demonstrate a decrease in dendritic arborization and axonal shortening^6^. Furthermore, modifications in neurotransmitters and effects on the distribution of neurofilament proteins also occur, implying changes in protein dysregulation and neuronal vulnerability. Cognitive changes in normal aging are associated with a decrease in the number of connections between neurons through a reduction in dendritic arborization, although not necessarily related to functional loss^6^.

From a clinical perspective, both macroscopic and microscopic alterations interfere with and impact cognitive abilities^7^. During the aging process, skills associated with fluid competencies tend to decline^8,9^, potentially affecting attentional span (aged individuals may struggle to select one stimulus over others or focus on multiple things simultaneously), working memory (simultaneous information manipulation), and executive functions^7-9^. Concerning executive functions, aged individuals may develop different decision-making strategies, tending to keep choices within the limits of their prior knowledge on the subject, rather than considering new information. There is a decline in cognitive flexibility, associated with operational capacity, planning, and the implementation of strategies. Age affects cognitive tasks that require complex mechanisms such as planning, organizing, and putting one or more strategies into action^7-9^. Therefore, the assessment of working memory should consider the aging process, since it is mediated in dorsolateral portions of the prefrontal cortex, and the aged may take longer to perform simultaneous tasks due to high demand^7-9^. This occurs due to reduced attentional resources, slowed information processing speed, and failure in inhibitory control mechanisms^7-9^.

Understanding the importance of neuropsychological assessment in the aged, particularly as a tool contributing to the diagnostic investigation of dementia syndromes and considering the decrease in fluid abilities in old age, the objective of this study was to present validity evidence for FDT in the evaluation of working memory.

## METHODS

Cross-sectional analysis involving 167 community-dwelling subjects attending a Gerontology Outpatient Clinic, conducted from February 2021 to November 2023. A total of 67 patients were removed from the study after the exclusion criteria were applied. The study included 100 educated participants, categorized into two groups: those with basic education (1 to 8 years of schooling) and those with high education (9 or more years of schooling). All participants underwent thorough clinical history and neuropsychological assessments, all of which were administered by the outpatient clinic’s neuropsychologist. We received the patients at the Gerontology Centers of Jundiaí, located in the southeastern region of Brazil, in São Paulo state. This city is ranked 12^th^ on the human development index^10^.

In the initial phase, participants underwent thorough clinical history with a geriatrician, including laboratory and neuroimaging examinations. In a subsequent phase, with a one-week interval following the clinical history, behavioral, psychiatric, and functional data were collected through clinical assessments involving the patient, as well as their relatives and caregivers. After this interview, aged individuals underwent neuropsychological assessment using the memory subtests of the Montreal Cognitive Assessment (MoCA-MIS)^11^, which assesses immediate and delayed recall memory. Additionally, FDT, evaluating attention, inhibitory control, working memory, and processing speed^4^, and the Digits subtest of the Wechsler Adult Intelligence Scale third edition (WAIS-III) and its sub-scores — Direct Order (DO), Indirect Order (IO), Digits Forward Span (SpanD), and Digits Backward Span (SpanI) — were employed to assess attentional performance and contribute to associative parameters of working memory^12^. Only MoCA was used to assess cognitive impaired performance. Other instruments were not used to stablish the diagnosis, which was determined after clinical, laboratory, and neuroimaging analysis.

### Inclusion and exclusion criteria

Inclusion criteria comprised men and women aged 50 years old or older, with an educational background (>1 year of schooling, indicating completion of at least the first year of primary education, currently referred to as Elementary School, Cycle I), willingness to voluntarily participate in the study, and the ability to sign the informed consent form. Inclusion criteria for the sample composition were as follows:

- Participants met dementia criteria in consonance with the revised Diagnostic and Statistical Manual of Mental Disorders (DSM-5-TR)^13^;
- Present impairment or limitations in functional activities based on the Pfeffer Functional Activities Questionnaire (FAQ) score (>5 points)^14^;
- Do not present depressive symptoms based on Geriatric Depression Scale (GDS) score (<5 points)^15^.

Exclusion criteria were previously established and applied to patients with severe dementia (Clinical Dementia Rating above 3.0)^16^, those exhibiting paralysis in both hands during the neuropsychological assessment, individuals showing signs of resting tremor and muscle stiffness indicative of parkinsonism at the time of evaluation, participants experiencing visual and auditory difficulties during the assessment, those with functional impairments as described by themselves, and individuals who declined to participate in the neuropsychological tests. A total of 67 subjects were excluded based on these criteria. The study protocol was approved by the Research Ethics Committee under approval number C.A.A.E. 50681621.2.0000.5412.

The Control Group consisted of participants who reported subjective memory complaints but, after clinical and neuropsychological investigation, exhibited no cognitive or emotional affective impairment. The sample composition for the Control Group was as follows:

- Did not meet dementia criteria according to DSM-5-TR^13^;
- Did not present impairment in functional activities based on the PFAQ (<4 points)^14^;
- Did not present any depressive symptoms based on GDS score (<5 points)^15^.

### Procedures for the Five Digit Test

FDT is a test restricted to psychologists and aims to assess executive functions involving simultaneous manipulation of information, cognitive flexibility, inhibitory control, and information processing speed^4^. It has broad applicability across age groups (6 to 92 years) and typically takes 5 to 10 minutes to complete. The FDT consists of two main parts: simple processing (Reading and Counting) and complex processing (Choosing and Shifting). Reading involves the visual identification of numbers (simple processing); Counting requires participants to enumerate asterisks (counting the quantity); Choosing involves counting groups of digits with conflicting values (maintaining attention during counting despite interference from reading); inhibiting the tendency to read the numbers; Shifting tasks require participants to alternate between two operations involving reading the quantity of numbers and identifying a specific digit number indicated by a thicker border every fifth group of digits^4^.

The first part of the test is considered simple processing as it does not require intentional effort from patients. The second part involves complex processing, relying on controlled and conscious actions, which force the patient to mobilize a higher level of mental resources. To perform the analysis, it is necessary to record the time and number of errors in all four parts of the test (Reading, Counting, Choosing, and Shifting). Two additional scores can also be obtained: Response Inhibition and Flexibility^4^.

### Evaluation procedures for working memory

Working memory can be defined as the ability to manipulate simultaneous information over a short period, involving components such as the central executive, the phonological loop, the visuospatial sketchpad, and the episodic buffer^17^. The encoding phase involves receiving and encoding sensory information or data from the environment (central executive), which is analyzed by verbal (phonological loop) and non-verbal (visuospatial sketchpad) components. Storage is the phase where the encoded information is temporarily retained and manipulated^17^. This component of working memory allows data to be retained for a short period, usually ranging from a few seconds to minutes. Finally, the Retrieval Phase involves retrieving and utilizing the temporarily stored information for specific tasks, such as answering questions, solving mathematical calculations, or performing complex mental activities^17^.

Working Memory is often associated with higher cognitive abilities, such as selective attention, inhibitory control, and cognitive flexibility, meaning it occurs in the context of executive functions. It is an essential component for performing daily activities that require the manipulation and active processing of information. Unlike long-term memory, which focuses on storing information for a more extended period, working memory deals with temporary data necessary for immediate task performance. A widely used test for its assessment is the WAIS-III scale task, the Digits subtest, and its scores^12^.

### Statistical analyses

Analyses were performed using the Statistical Package for Social Sciences (SPSS) IBM^®^, version (25.0). Normality test was conducted through the Kolmogorov-Smirnov test. Mean differences were examined using ANOVA with the Tukey method. The correlation level of cognitive tests (FDT with Reading, Counting, Choosing, Shifting, and Inhibition items, as well as error numbers) with WAIS Digits (total score, DO, IO, SpanD, and SpanI) was assessed using Spearman’s correlation coefficient [rho], stratified by diagnostic group (control and experimental groups). Finally, the statistical program MedCalc (version 15.8) was used for sensitivity and specificity analysis between FDT Shifting and WAIS-III Digits total score, which involved Receiver Operating Characteristic (ROC) curve analysis.

## RESULTS

Outcomes regarding a total sample of 100 subjects with a mean age of 71.04 years (minimum= 56; maximum= 86; standard deviation [SD]=6.45), 61.5% (n=59) females, and an average education level of 67.3%, were divided into two groups: the control group (CG) and those diagnosed with cognitive impairment. The description of demographic data (age, gender, and education), as well as cognitive aspects of MoCA-MIS (immediate and delayed recall memory), FDT (Reading, Counting, Choosing, Shifting, Inhibition, and Flexibility), and Digits scores, divided by diagnostic group, are presented in Table 1. Statistically significant differences were observed in age, gender, and education, indicating that the control group was younger and more educated compared to the group with cognitive decline. Additionally, statistically significant differences were demonstrated for Immediate Memory (p<0.0001) and Delayed Recall (p<0.0001). Regarding FDT, notable differences were observed in complex tasks, particularly in Shifting time analysis (p=0.039) and number of errors (p=0.034). The Digit score (p=0.002) and its subtests also showed statistically significant differences between the two groups (Table 1).

**Table 1:** Mean and standard deviation of cognitive tests and percentage of age, schooling, and gender of 100 participants.

Characteristics	Control group	Cognitive decline	p-value
	Mean (SD) n=36	Mean (SD) n=64	
Age	67.92 (5.24)	73.11 (6.61)	**0.010**
Sex (%)			
Female	75 (n=27)	53.1 (n=34)	**0.025**
Male	25 (n=9)	46.9 (n=30)	
Schooling (%, years)			
<8	36.1 (n=13)	61 (n=39)	**0.025**
>9	63.9 (n=23)	39 (n=25)	
MoCA-MIS immediate	9.44 (0.94)	7.76 (1.86)	**0.0001**
MoCA-MIS delayed memory	13.25 (2.33)	8.44 (4.35)	**0.0001**
FDT reading (time)	27.23 (6.35)	34.48 (24.80)	0.054
FDT reading (errors)	0.34 (2.03)	0.60 (2.06)	0.072
FDT counting (time)	29.43 (7.22)	38.41 (32.12)	**0.037**
FDT counting (errors)	0.66 (3.39)	0.48 (1.95)	0.392
FDT choosing (time)	51.03 (17.59)	61.69 (59.61)	0.230
FDT choosing (errors)	2.29 (3.78)	4.55 (7.02)	0.303
FDT shifting (time)	68.31 (28.65)	85.93 (70.97)	**0.039**
FDT shifting (errors)	3.51 (3.82)	7.40 (7.70)	**0.034**
Inhibition	23.14 (13.77)	26.77 (58.53)	0.988
Flexibility	39.94 (25.02)	50.63 (68.04)	0.118
Digits score	13.06 (4.12)	10.41 (3.27)	**0.002**
DO	8.09 (2.38)	6.84 (1.79)	**0.024**
IO	4.97 (2.18)	6.19 (12.79)	**0.040**
Span digits forward	7.43 (2.08)	6.24 (1.97)	**0.020**
Span digits backward	4.06 (1.85)	3.32 (1.67)	**0.050**

Abbreviations: SD, Standard Deviation; MoCA-MIS, Montreal Cognitive Assessment; FDT, Five Digit Test; DO, Digits subtests in direct order; IO, Digits subtests in indirect order. Notes: Age p-value=Tukey, Gender, and Schooling p-value=χ^2^; Cognitive Testes p-value=Mann-Whitney. Note: Bold indicates statistically significant p-values.

To address the objectives of this research, which aimed to explore FDT’s working memory, the association between groups was analyzed using the Spearman’s correlation coefficient, as described in Table 2. Moderately positive and significant correlation coefficients were observed between FDT Reading (time) and age (rho= 0.30; p=0.003) and Reading (errors) and age (rho=0.30; p=0.004). Moderately negative and significant correlation coefficients were found between the FDT Reading (time) and Digit score (rho=-0.51; p<0.0001), DO (rho=-0.46; p=0.0001), IO (rho=-0.37; p<0.0001), and SpanD (rho=-0.43; p<0.0001). FDT Counting time analysis also showed correlation coefficients with Digits analyses, as shown in Table 2. However, what draws more attention is the number of errors in the complex tasks of FDT and WAIS-III Digits. FDT Choosing (errors) exhibits a moderately negative and significant correlation with Digit score (rho=-0.31; p=0.002), IO (rho=-0.41; p<0.0001), and SpanI (rho=-0.32; p=0.002). FDT Shifting (errors) presented the most robust data, with a moderately negative and significant correlation with Digit score (rho=-0,51; p<0,0001), DO (rho=-0.39; p<0.0001), IO (rho=-0.46; p<0.0001), SpanD (rho=-0.34; p<0.001), and SpanI (rho=-0.38; p<0.0001).

**Table 2:** Spearman correlation coefficient of 100 participants between Five Digit Test and Digits and their subtests.

Characteristic		Age	Digit score	DO	IO	SpanD	SpanI
FDT reading (time)	*rho*	**0.30**	**-0.51**	**-0.46**	**-0.37**	**-0.43**	-0.25
	p	**0.003**	**0.0001**	**0.0001**	**0.0001**	**0.0001**	0.016
FDT reading (errors)	*rho*	**0.30**	-0.25	-0.21	-0.22	-0.19	-0.27
	p	**0.004**	0.014	0.045	0.032	0.068	0.009
FDT counting (time)	*rho*	0.15	**-0.48**	**-0.43**	**-0.38**	**-0.41**	-0.28
	p	0.135	**0.0001**	**0.0001**	**0.0001**	**0.0001**	0.006
FDT counting (errors)	*rho*	0.08	-0.15	-0.12	-0.13	-0.12	-0.19
	p	0.463	0.149	0.248	0.223	0.249	0.066
FDT choosing (time)	*rho*	0.19	**-0.45**	**-0.38**	**-0.38**	**-0.32**	-0.22
	p	0.057	**0.0001**	**0.0001**	**0.0001**	**0.001**	0.032
FDT choosing (errors)	*rho*	0.18	**-0.31**	-0.20	**-0.41**	-0.17	**-0.32**
	p	0.077	**0.002**	0.059	**0.0001**	0.107	**0.002**
FDT shifting (time)	*rho*	0.24	**-0.50**	**-0.43**	**-0.40**	**-0.41**	-0.27
	p	0.018	**0.0001**	**0.0001**	**0.0001**	**0.0001**	0.008
FDT shifting (errors)	*rho*	0.27	**-0.51**	**-0.39**	**-0.46**	**-0.34**	**-0.38**
	p	0.035	**0.0001**	**0.0001**	**0.0001**	**0.001**	**0.0001**
Inhibition	*rho*	0.06	-0.20	-0.15	-0.24	-0.11	-0.11
	p	0.544	0.044	0.154	0.017	0.282	0.282
Flexibility	*rho*	0.15	**-0.35**	-0.28	-0.28	**-0.30**	-0.20
	p	0.149	**0.001**	0.005	0.005	**0.004**	0.052

Abbreviation: DO, direct order; IO, indirect order.

Notes: Significant moderate correlations are highlighted in bold; Underlined indicates significant but weak correlation coefficients.

To fulfill the main objective, the ROC curve methodology was employed. Table 3 ^18^ describes the sensitivity and specificity data for instruments that showed the most moderately significant correlation coefficients. For this purpose, FDT number of errors in Choosing and Shifting, as well as Digits and its subtests, were selected. The results indicate that the instrument with the largest Area Under the Curve (AUC) was FDT Shifting (errors) (AUC=0.935), followed by Digit score (AUC=0.748) and FDT Choosing (errors) (AUC=0.730). For FDT Shifting (errors), the cutoff point for identifying cognitive impairment was >5 points.

**Table 3:** Analyses of the size of difference of curves^18^.

Characteristics	AUC	SE	95%CI*	Sen. (%)	Spe (%)	Cutoff point	p-value
FDT shifting (errors)	0.935	0.935	0.863 to 0.976	80	95	>5	0.0001
Digit score	0.748	0.0507	0.648 to 0.831	78	65	≤11	0.0001
FDT choosing (errors)	0.730	0.0523	0.626 to 0.818	65	71	>1	0.0001
IO	0.722	0.0517	0.620 to 0.810	77	55	≤4	0.0001
SpanI	0.693	0.0543	0.589 to 0.785	52	79	≤2	0.0004
DO	0.677	0.0549	0.573 to 0.770	73	59	≤7	0.0013
SpanD	0.670	0.0545	0.566 to 0.764	82	46	≤7	0.0018

Abbreviations: AUC, Area under the curve; SE, standard error; 95%CI, 95% confidence interval; Sen, sensitivity; Spe, specificity; p-value, significance level; FDT, Five Digit Test; IO, indirect order; SpanI, digits backward span; DO, direct order; SpanD, digits forward span.

Notes:*Binomial exact.

**Figure 1:**
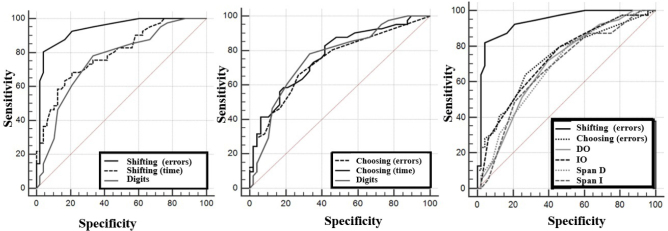
Receiver Operating Characteristic Curve Methodology in relation to cognitive functions: Five Digit Test (time and errors) and digits.

**Figure 2:**
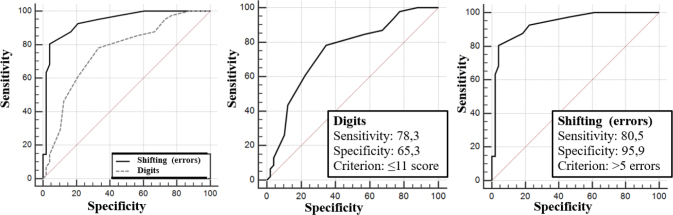
Receiver Operating Characteristic Curve Methodology in relation to Shifting, number of errors, and total score of digits from WAIS-III.

Figure 1 demonstrates the ROC curve methodology for cognitive instruments performance. To better illustrate the evidence of working memory validity through FDT Shifting (errors), Figure 2 describes the comparison between its results and the comparison with Digits score. A higher AUC is observed in FDT Shifting (errors), indicating satisfactory psychometric properties for identifying cognitive deficits compatible with working memory impairment in the aged, especially those with cognitive impairment. FDT Shifting (errors) showed satisfactory results with a sensitivity of 80.5% and specificity of 95.9% for a cutoff point of >5 errors. Digits score was the second instrument with the highest AUC, with a sensitivity of 78.3% and specificity of 65.3%, for a cutoff point ≤11 points. The data suggest that the diagnostic investigation of older individuals with cognitive impairment and working memory deficits can be done through the FDT Shifting number of errors instrument, suggesting that errors greater than four points may indicate working memory impairment (Figure 2).

## DISCUSSION

The aim of this study was to present sensitivity and specificity data (validity evidence) for the FDT test in Brazilian aged individuals, particularly in the construct of working memory. FDT is a neuropsychological test with broad applicability and importance in investigating executive functions. However, its clinical practicality has shown that the number of errors in complex tasks may indicate impairment in working memory. The results demonstrated that the healthy aged group appeared younger and had more years of education, corroborating findings in the literature on dementia prevention, especially related to years of education^19-22^.

Studies have shown a correlation between FDT and the Digits subtest of the WAIS-III with satisfactory psychometric properties. In a study with Brazilian aged individuals, older participants (over 80 years) with complaints of depressive symptomatology exhibited poorer performance in FDT Inhibition and FDT Flexibility compared to the younger aged group, as well as in the Digit Span Indirect performance^22^. In a study conducted by Chagas et al.^23^, FDT was used for the validation of a cognitive screening and correlated significantly with executive function performance. Our study aligns with literature data on significant correlations regarding the applicability of FDT in the diagnostic investigation of aged individuals^23-25^. Another study highlights the importance of FDT in the Brazilian population, investigating FDT performance among individuals aged 18 to 65, demonstrating the fundamental relevance of sustained attention and information processing speed^24^. Paiva et al.^24^ presents average times for FDT in the studied population with Reading 22.67 seconds, Counting 25.43 seconds, Inhibition 15.97 seconds, and Flexibility 24.82 seconds. When compared to our findings, it is evident in Table 1 that the time results in this research are significantly higher than in the study by Paiva et al.^24^. One justification for this difference is related to age. With advancing years, there is a significant decline in fluid abilities in old age^8,9^, which may account for the longer execution time, as Paiva et al.^24^ analyzed individuals between 18 and 65 years old, while our subjects had an average age of 71.04 years.

What stands out in our results is the number of subjects (n=100) as well as the analysis of the number of errors in the FDT. To our knowledge, this study is one of the few that aimed to analyze the number of errors in the FDT in the aged population. Saffi et al.^25^ conducted a study with 72 participants, both healthy and with mental disorders, comparing the number of errors in the FDT with other cognitive instruments. However, the research group divided the number of errors into two parts: the first part in automatic processes corresponding to FDT Reading and FDT Counting, and the second part, which they termed controlled processes, corresponding to FDT Choosing and FDT Shifting. The present study proposed to divide all errors into the four tasks of the FDT (Reading, Counting, Choosing, and Shifting) and present evidence of validity mainly for FDT Shifting, which demonstrated that a cutoff point of 5 or more errors may indicate cognitive impairment. Furthermore, as described in the methodology of this research, FDT Shifting involves the recall of information, meaning that the examinee needs to remember the rule to perform the task satisfactorily. Thus, it can be inferred that the execution of FDT Shifting requires the involvement of brain structures in the frontal lobe that regulate simultaneous information manipulation with rule recall for correct execution. With this definition, it can be verified that FDT Shifting contributes to the analysis of working memory by its design. Moreover, the moderate and significant correlation between this construct and Digit score (Table 2) demonstrates evidence of validity between the two cognitive tests.

In conclusion, our contributions consist of suggesting the adoption of the FDT Shifting number of errors as a neuropsychological assessment for aged patients, especially beneficial for investigating impairment in patients suspected of dementia. For this instrument, we adopted a cutoff threshold of five or more errors for this measure. In addition, we aim to broaden the scope of FDT analysis to include assessments of working memory.
